# Towards the Generation of Medical Imaging Classifiers Robust to Common Perturbations

**DOI:** 10.3390/biomedinformatics4020050

**Published:** 2024-04-01

**Authors:** Joshua Chuah, Pingkun Yan, Ge Wang, Juergen Hahn

**Affiliations:** 1 Department of Biomedical Engineering, Rensselaer Polytechnic Institute, Troy, NY 12180, USA; 2 Center for Biotechnology and Interdisciplinary Studies, Rensselaer Polytechnic Institute, Troy, NY 12180, USA

**Keywords:** machine learning, artificial intelligence, medical imaging, robustness

## Abstract

Background: Machine learning (ML) and artificial intelligence (AI)-based classifiers can be used to diagnose diseases from medical imaging data. However, few of the classifiers proposed in the literature translate to clinical use because of robustness concerns. Materials and methods: This study investigates how to improve the robustness of AI/ML imaging classifiers by simultaneously applying perturbations of common effects (Gaussian noise, contrast, blur, rotation, and tilt) to different amounts of training and test images. Furthermore, a comparison with classifiers trained with adversarial noise is also presented. This procedure is illustrated using two publicly available datasets, the PneumoniaMNIST dataset and the Breast Ultrasound Images dataset (BUSI dataset). Results: Classifiers trained with small amounts of perturbed training images showed similar performance on unperturbed test images compared to the classifier trained with no perturbations. Additionally, classifiers trained with perturbed data performed significantly better on test data both perturbed by a single perturbation (*p*-values: noise = 0.0186; contrast = 0.0420; rotation, tilt, and blur = 0.000977) and multiple perturbations (*p*-values: PneumoniaMNIST = 0.000977; BUSI = 0.00684) than the classifier trained with unperturbed data. Conclusions: Classifiers trained with perturbed data were found to be more robust to perturbed test data than the unperturbed classifier without exhibiting a performance decrease on unperturbed test images, indicating benefits to training with data that include some perturbed images and no significant downsides.

## 1. Introduction

Medical imaging modalities such as X-ray, CT, and MRI are often used as a non-invasive procedure to obtain physiological data [1]. Frequently, clinicians use these data for the early detection and diagnosis of diseases such as cancer and certain cardiovascular conditions [2,3]. However, variations in the pathology of diseases and the physiology of patients can make it difficult for human clinicians to reliably deliver accurate diagnoses [4]. Additionally, although advancements in technology have made medical imaging data more widely available, their development has resulted in more complex datasets [5,6]. To address these issues, extensive research has gone into the creation of computer-aided diagnosis (CAD) tools to improve the quality of diagnoses by supporting the clinician’s decision-making process [7,8].

CAD tools often implement artificial intelligence (AI) or machine learning (ML) models for their ability to learn patterns from data [9]. An AI/ML classifier is a model that can be trained using previously collected clinical data to predict whether data from a new sample indicate a positive or negative diagnosis. While AI/ML diagnostic classifiers have achieved good performance for diagnosing certain diseases in the literature, they have not seen widespread clinical implementation [10–12]. This is partially because it has been shown that AI/ML classifiers can perform poorly when the information contained within the data is complex, i.e., if the data are heterogeneous or noisy [13]. This is especially problematic as differences in techniques, clinical practices, and testing equipment can all be sources of noise in a dataset.

There have been many case studies demonstrating improved reliability for specific classifiers for certain applications. Mardani et al. developed a generative adversarial network (GAN) with an affine projection operator to remove aliasing artifacts from MRI images. This model was shown to be robust to even extreme forms of Gaussian noise [14]. Janizek et al. developed a model that improved generalization between clinics by alternating training between an adversary attempting to predict adversarial samples and a classifier trying to fool the adversary [15]. Another application to cancer MRI images used deep feature extraction to find a feature set that achieved high performance across 13 convolutional neural networks and was generalizable [16]. Gulzar and Khan were able to use a TransUNet model for a skin cancer segmentation model robust to distortions [17]. Further research has used adversarial defense methods such as MedRDF, non-local codex encoder (NLCE) modules, and kernel density adversary detection to improve the robustness of deep medical imaging classifiers to adversarial noise [18–20]. There are no widespread guidelines used to evaluate model robustness [21–23]. Therefore, the robustness of classifiers, i.e., the ability that allows translating the predictions to new, potentially noisy, data, remains a barrier to entry for the clinical implementation of AI/ML diagnostic models.

Previous studies have shown that even classifiers with good performance can lack robustness [24,25]. This lack of stability amongst classifiers has led to increased efforts to characterize the robustness of imaging-based classifiers. To this end, many researchers have focused on creating “benchmark sets” or test sets that contain perturbed/augmented images [26–28]. Robustness is then typically measured by how well a given classifier, usually trained with relatively low-noise data, predicts the benchmark set images. Recently, studies have shown that adversarial training or data augmentation can potentially improve the robustness of classifiers applied to medical imaging studies [29,30]. One example of this is ROOD-MRI, which enables researchers to evaluate the sensitivity of their MRI segmentation model to out-of-distribution and corrupted samples [31]. Another example is CAMELYON17-WILDS, which serves as a benchmark set for in-the-wild distribution shifts, e.g., heterogeneity between hospitals, for brain tumor segmentation [32]. Furthermore, benchmarks have been developed to evaluate the adversarial robustness of classifiers pertaining to skin cancer classification and chest X-ray images [26,29].

While data augmentation has been effectively used to increase classifier robustness to perturbation, there are still gaps in the literature that prevent these from being used for diagnostic tests [33]. First, to the best of the authors’ knowledge, there is no research that explores the effect on a classifier’s robustness when different amounts of the training/test images are perturbed simultaneously. As such, dataset heterogeneity such as measurements at multiple clinical sites or using different equipment is often ignored. Second, most studies add perturbed images to the training set with the goal of improving classifier robustness, rather than replacing the original images to determine how the classifier changes what it learns [34,35]. Finally, there is only very limited research regarding multiple perturbations, and none that addresses the scenario where not all images receive the same combination of perturbations [36]. To fill this gap, the contributions of this study are to (1) show that deep learning classifiers trained with medical images augmented by common perturbations can improve performance on perturbed images without necessarily sacrificing performance on unperturbed data, (2) demonstrate a perturbation scheme that allows researcher to evaluate the extent to which a classifier is robust to perturbed data, and (3) determine if classifiers trained with sets of different perturbations will perform well on images simultaneously perturbed by multiple perturbations.

The next section (Section 2) describes the details of the procedure used in this study, as well as the datasets and types of noise used. Section 3 presents the results of applying this procedure to two example datasets. Section 4 discusses the results and interprets the findings. Finally, Section 5 reflects on the overall implications of this study.

## 2. Materials and Methods

The next Section 2.1 proposes the general classifier training/testing procedure for a given classifier, dataset, and set of perturbations. Section 2.2 explains the perturbations used in this study, and Section 2.3 introduces the different datasets used in this study. Finally, these are combined in Section 2.4, which shows how the general outline of the training/testing scheme was applied to find classifiers trained with specific datasets on the chosen perturbations.

### 2.1. Classifier Training/Testing Procedure

The goal of this study is to develop a procedure for training a robust classifier given a dataset in the form of medical images. Such a procedure must evaluate a classifier’s (1) performance on unperturbed test data to determine baseline performance and (2) performance on test data with different amounts of perturbed data to determine how stable a classifier is when the data are noisy and (3) the consistency of the performance. In this study, a classifier is deemed robust to a perturbation if its performance on unperturbed data is similar to perturbed data. This section provides a general description of the developed procedure. The presented procedure is then tested on a suite of perturbations, explained further in Section 2.2, and applied to an example dataset, presented in Section 2.3, and a more detailed discussion of this application is provided in Section 2.4.

In the first step, a classifier *f* is developed on the training set with no additional perturbations added (i.e., the clean dataset). This classifier serves as a baseline classifier to identify whether potential performance loss is due to perturbing the training set or test set. Using the same network architecture (but re-training the model weights), a second classifier *f*_*p*_^*n*^ is trained on the training data with a randomly selected percentage, *p*%, of the images perturbed by perturbation type *n* (e.g., Gaussian noise). Several classifiers are trained and tested this way using various values of *p* and different perturbations. These classifiers are then tested on the unperturbed test set images. Training classifiers with varying *p* is important as the amount of perturbed data can greatly impact classifier behavior. A classifier that is robust to a perturbation will exhibit similar behavior to a classifier trained with some data that have been perturbed by that perturbation. However, satisfying this criterion alone does not necessarily mean a classifier is robust, as it must also perform well when there are perturbations, even of other types, in the test set.

**Figure 1.**
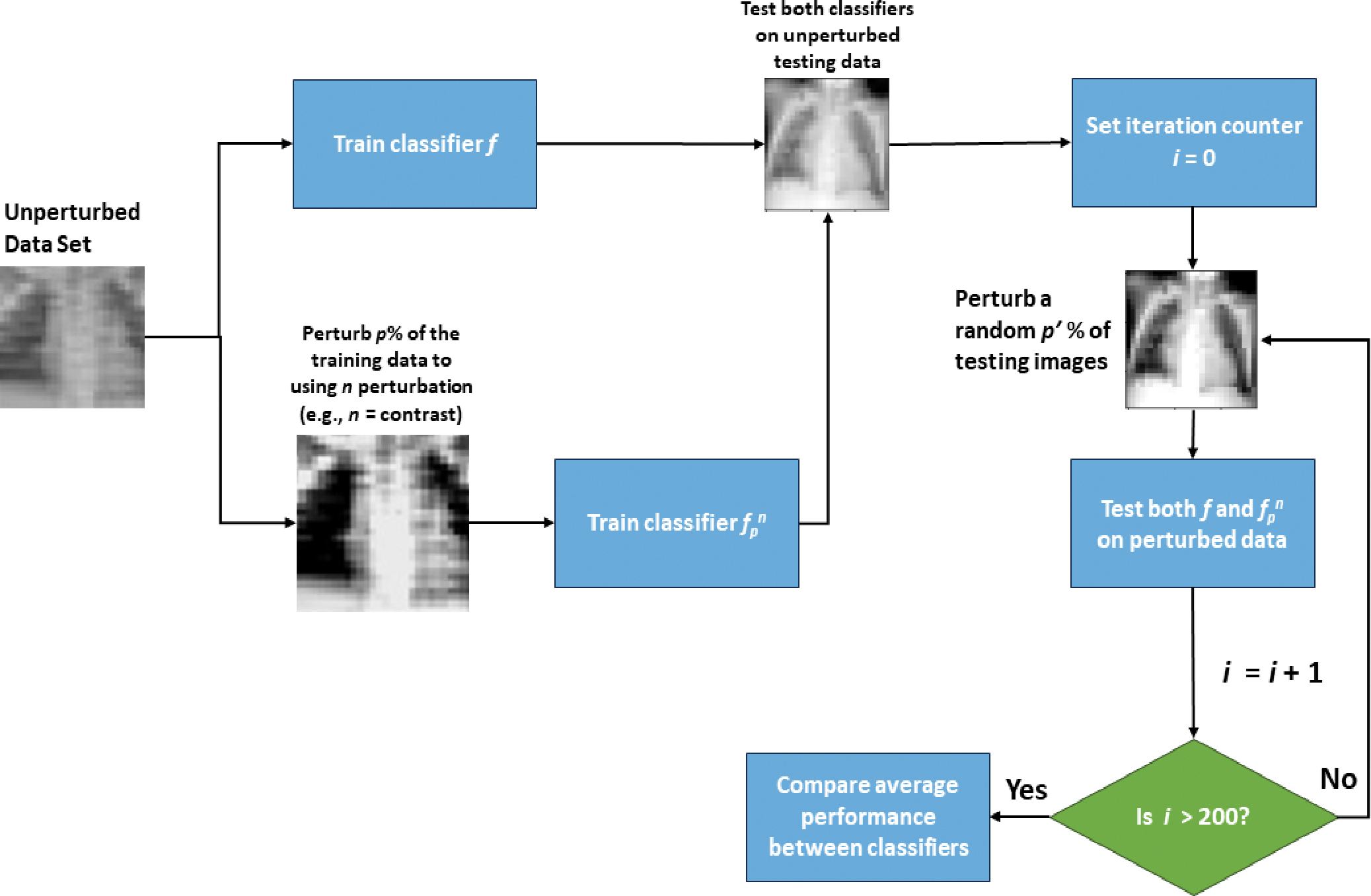
Basic flowchart of the classifier perturbation and testing process.

The goal of testing the chosen classifiers with some portion of perturbed data is ultimately to determine if a classifier is learning the original data, the noise, both, or neither; although, in most cases, it is a combination of these. To accomplish this goal, the already-trained classifiers from the previous step are evaluated using test data with various amounts of images perturbed. Similar to the previous step, a random *p*′ percent of the images in the test set are perturbed using perturbation *n*. Differently, this step makes use of repeatedly perturbing the test data to evaluate classifier performance. For each value of *p*′ and *n*, multiple test sets are generated such that a different (random) selection of *p*′% images are perturbed in each set. The performance of a classifier for that combination of *p*′ and *n* is then recorded as the average performance over all repeatedly perturbed test sets. Averaging is needed if the performance varies greatly amongst these test sets. Additionally, if *f*_*p*_^*n*^ performs similarly to *f* on the unperturbed test data and outperforms *f* on the perturbed test data, the classifier *f*_*p*_^*n*^ is likely more robust than *f* to perturbation *n*. A walkthrough describing the steps of this workflow is shown in Figure 1.

### 2.2. Perturbations

**Figure 2.**
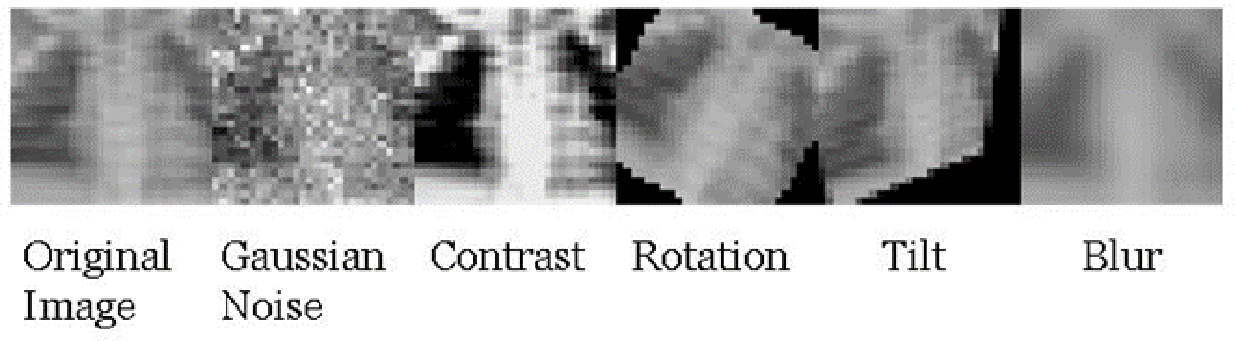
Example of how the perturbations used in this study alter the original image.

Five commonly seen perturbations were applied to the data in this study: Gaussian noise, contrast, rotation, tilt, and blur. An example of these applied to an image is shown in Figure 2. A brief description of the perturbations used is included in Table 1. Gaussian noise was applied by adding or subtracting a number randomly sampled from an N (0, *s*^2^) distribution [37]. The value of *s* is directly proportional to the severity of the Gaussian noise in the image. In this study, *s* = 0.08, which is the minimum value employed by Dietterich and Hendrycks (pixel values are scaled down to a range of 0–1, noise is applied, then rescaled to the original data scale of 0–255) [27]. This value was chosen as it was important to see if any Gaussian noise would cause a change in classifier performance, as it is expected that one would see a drop in performance if the applied noise is severe. Contrast adjustment was applied by using Haeberli and Voorheis’ extrapolation method implemented in the Python Imaging Library to create similar effects as in Hendrycks and Dietterich’s code [38]. The rotation perturbation was applied by rotating the entire image on the xy-plane. The rotation of each image was a randomly selected angle between −90 and 90 degrees. The tilt perturbation was applied by making a section of the image smaller as if it were pushed down the *z*-axis. The tilt of each image was a randomly selected angle between 0 and 90 degrees. The range of possible angles for rotation and tilt perturbation were enlarged to allow for more severe perturbations. Blur was applied by applying the opencv GaussianBlur function according to Dietterich and Hendrycks, i.e., using the same severity [27]. Blurring of images was applied at the lowest severity that was tested in their study. While perturbations like noise and rotation were applied randomly to each image, the severity of noise was not varied in this study. While different perturbation intensities were not used in this study, the intensity of each of the implemented perturbations was measured by computing the average structural similarity index metric (SSIM) among 200 test sets fully replaced with perturbed images. It should be noted for future studies employing this strategy that changing the severity of each perturbation, such as the maximum rotation angle or standard deviation of the Gaussian noise distribution, may potentially lead to different performance.

Although it was not one of the main perturbations being observed, adversarial noise was still used in this study. Adversarial attacks are image perturbations or corruptions that are specifically tailored to cause a classifier to misclassify an image without making the image look perceptibly different to the human eye [39]. These differ from the other perturbations used in this study, as they require details about the network structure and current classification of the images to successfully perturb the image. In this study, the simultaneous perturbation stochastic approximation (SPSA) method was used to perform adversarial training for a classifier [39]. Rather than using the gradient to indicate the optimal adversarial attack application to cause a misclassification, SPSA is a gradient-free method that approximates this gradient using finite differences in random directions [39]. The SPSA adversarial attack was implemented using the Cleverhans Python package implementation [40].

### 2.3. Datasets

Two datasets were used for this study. One is the publicly available PneumoniaMNIST dataset [41,42], while the other one is the Breast Ultrasound Images (BUSI) dataset [43]. The PneumoniaMNIST dataset contains chest X-ray images of patients who either had a positive or negative pneumonia diagnosis. Images were collected from children aged 1–5 in JPG format. Images were labeled by the evaluation of two expert physicians. Further information can be found in [42]. The PneumoniaMNIST data are a processed version of Kermany and Goldbaum’s originally published dataset where the images were compressed to be 28 × 28. This was performed primarily to save computational time. The PneumoniaMNIST dataset is composed of 5856 images, of which 4708 are reserved for the model training set, 524 are used for model validation, and 624 are used for the model test set. These sets were assigned by the MedMNIST database as part of the preprocessing step [41]. Approximately 74.2% of the training and validation images and 62.5% of the test images were pneumonia-positive samples (label = 1), with the rest being pneumonia-negative (label = 0).

The Breast Ultrasound Images dataset was also used to determine if perturbing larger images would have an impact on classifier performance [43]. This dataset is composed of breast ultrasound images containing benign, malignant, and no tumors. For binary classification purposes, benign and malignant images were considered part of the same group (containing a tumor), with the other group being the images without a tumor. The images used in this dataset were collected from women ages 25–75 and are in PNG format. Labels were assigned to the image by the physicians’ evaluation. Further information can be found in [43]. There were 779 images used from this dataset; 545 were assigned to the training set; 78 were assigned to the validation set; 156 were assigned to the test set. All sets contained the same ratio of tumor images to non-tumor images, approximately 82.6%. All images were resized to be 500 × 500 from their original sizes, as this was the average size of the raw images. This dataset was only used for the analysis comparing multiple randomly applied perturbations.

### 2.4. Experimental Protocol

**Figure 3.**
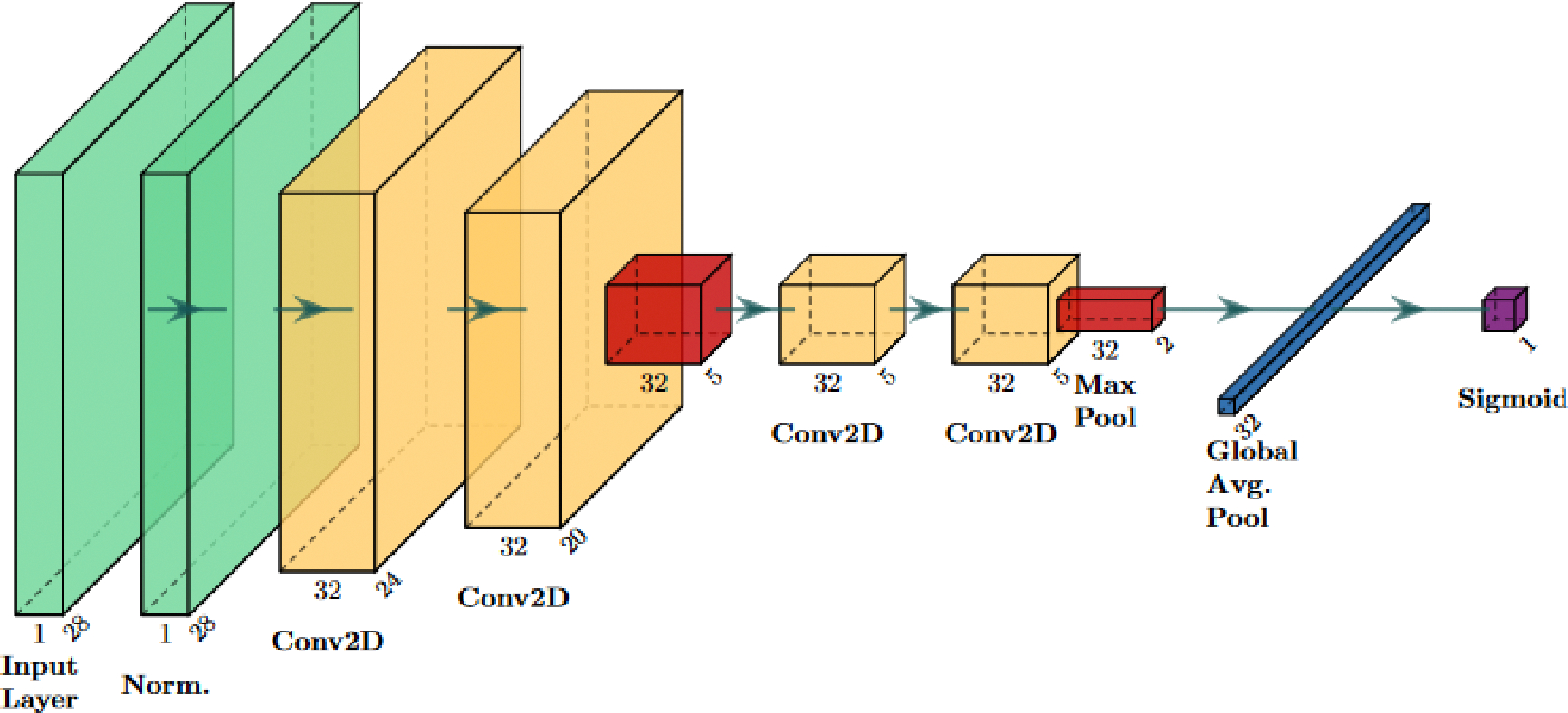
Diagram for one of the 10 neural network architectures used in this study. This architecture corresponds to network 7 in Table S1. Green layers are the input layers; yellow layers are the convolutional layers; orange layers correspond to the max pooling layers; blue layers are the flattened/vector layers; the purple layer is the activation head. The “CastToFloat32” and “ExpandLastDim” layers were omitted as they do not have any parameters and do not transform the base data [46].

This section details an application of the general method outlined in Section 2.1 by applying the perturbations detailed in Section 2.2 to the data introduced in Section 2.3. Ten deep learning classifiers (artificial neural networks) were trained in this study to determine the general performance trends of deep medical imaging classifiers when data are perturbed, rather than evaluating a single neural network architecture. For example, the reported performance of *f*_20_^*blur*^ is the average of the performance of 10 classifiers trained with 20% blur-perturbed training images. In this way, the results were not dependent on a specific classifier architecture and could be generalized to slightly different neural network architectures. Each classifier was a convolutional neural network trained using the AutoKeras neural architecture search algorithm [44]. Neural architecture search algorithms, given an overall architecture, try to find the setting of nodes and layers that maximize classifier performance [45]. Generally, AutoKeras works by allowing the user to input “blocks”, which include families of layers from the model to choose from in a specific order. For this experiment, all networks included an input block, a convolution block, and an output block. The input block contains preprocessing transformations of the images, which are meant to improve performance. The convolution block means the next few layers are all convolution layers of different sizes depending on the neural network architecture search seed. Finally, the model ends with a series of layers to convert the data into a one-dimensional layer that can be fed through a sigmoid activation layer, enabling binary classification. A diagram showing one of these neural network architectures can be found in Figure 3. The exact sequence of layers in each neural network trained on the PneumoniaMNIST dataset can be found in Table S1, and the architecture of the neural networks trained on the BUSI dataset can be found in Table S2.

**Figure 4.**
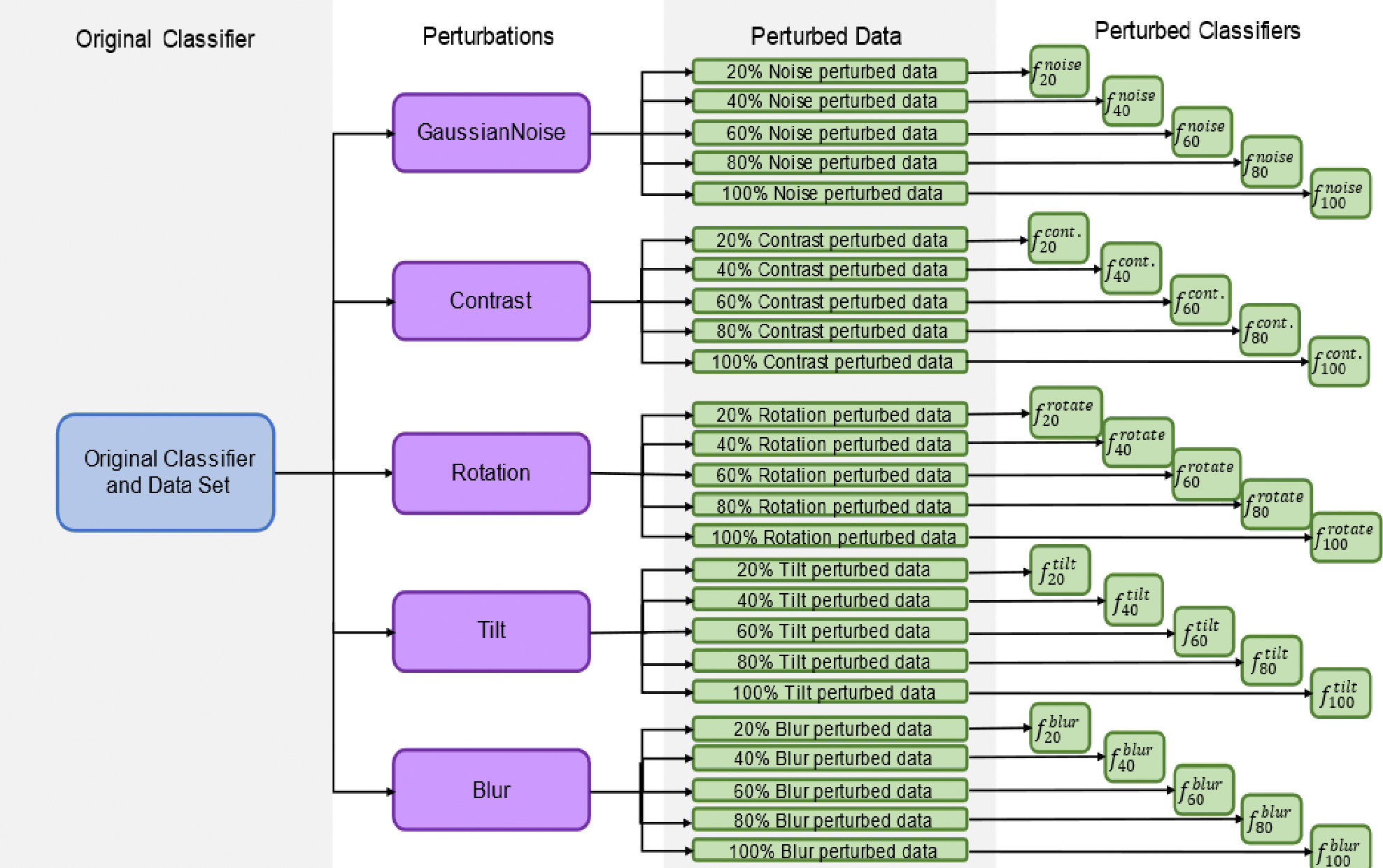
Flowchart for the creation of all perturbed classifiers based on an original classifier and a dataset.

The classifiers were first trained on the unperturbed training data to obtain the network architecture and model weights. Then, new classifiers were trained on the training data, where 20% of the data had been perturbed by each classifier (e.g., classifiers *f*_20_^*blur*^, *f*_20_^*contrast*^, etc., were trained). Images perturbed this way replaced their original (unperturbed) counterparts in order to keep the training set sample size constant throughout. To traineach classifier on the perturbed data, the model weights of the original network *f* were re-trained without changing the network architecture (weights were shuffled, then re-trained). This training was repeated in intervals of 20% (40%, 60%, etc.) to generate a total of 26 classifiers per network architecture/given classifier (i.e., 260 total for this study), 1 for each combination of *p* (20%, 40%, 60%, 80%, 100%) and *n* (Gaussian noise, contrast, tilt, blur, rotation) plus the unperturbed classifier *f*. A step size of 20% was chosen for perturbation to understand the general behavior of the classifiers as more data were perturbed without being too computationally exhaustive. Each of these classifiers was then evaluated by predicting the unperturbed test set data. The process of training these classifiers is shown in Figure 4.

Classifier performance was also evaluated on perturbed test set images. Similar to perturbing the training set data, the test set data were also perturbed in increments of 20% of the number of images in the test set. For each combination of *p*′ and *n*, 200 test sets were generated. These test sets were generated such that randomly selected *p*′ test images were perturbed using perturbation *n* each time. For each combination of *p*′ and *n*, the performance was the average error rate of a classifier over all 200 test sets. While other metrics are useful for assessing a classifier’s performance, this study is primarily concerned with diagnostic performance (e.g., accuracy), and therefore, the error rate, or the inverse of the accuracy, was used as the primary metric. Details on the precision and recall results are included in the Appendix A. The error rate for a given classifier on a set of images can be calculated by Equation (1) [47]:

(1) $$Error\;Rate=1-\frac{\text{Number}\;\text{of}\;\text{correct}\;\text{predictions}}{\text{Total}\;\text{number}\;\text{predictions}}$$

The error rate is effectively the proportion of misclassified test set images, e.g., an error rate of 0.15 indicates that the classifier misclassified 15% of the test set images. As such, an error rate close to 0 is preferable, and an error rate close to 0.5 indicates random predictions by the classifier. The perturbed classifiers used for robustness evaluation were all classifiers trained on 20%, 60%, and 100% perturbed data to reflect what happens when a small amount of the data are perturbed (20%), the majority of the data are perturbed (60%), and all of the data are perturbed (100%). The classifiers trained with 40% and 80% of the data were not used for further analysis because their behavior did not differ greatly from the other 3 scenarios (20%, 60%, and 100%), while also requiring significantly more computational resources to test. If a classifier was perturbed by a certain *n*, it was only tested using test images perturbed by the same perturbation. The performance (error rate) of each of these classifiers was compared to *f*, the scenario where no data were perturbed.

As a control measure to determine that any changes in performance were perturbation-specific, a classifier was also re-trained where all images were perturbed by simultaneous perturbation stochastic approximation (SPSA) adversarial noise with an intensity of 8/255 [20,48]. In theory, this classifier should have similar performance to the unperturbed classifier, since the classifier was still trained on a different distribution than was seen in the perturbed test data. A comparison of these error rates is presented in Section 3.

Although examining the impact of individual perturbations on classifier robustness is important, in reality, images are likely subjected to multiple simultaneous perturbations, and any robust classifier needs to take this scenario into account. To simulate this scenario, a new classifier was trained based on training data for each of the 10 generated neural network architectures, where 10% of the samples were perturbed by each perturbation type, leaving 50% of the training data unperturbed. Then, for each test set image, each perturbation was given a 50% chance of being applied to that image. In essence, each image was randomly assigned perturbations and could be perturbed by anywhere from 0 (none) to 5 (all) of the perturbations. Two hundred multiple randomly perturbed test sets were generated this way to better approximate the general behavior of each classifier. Each unperturbed classifier (10 architectures) and each perturbed classifier (each of the 10 architectures trained on the new training data) were evaluated by obtaining the error rate on each of the multiple randomly perturbed test sets. This was repeated for both datasets to determine if training with a portion of a perturbation alone could cause the classifier to be more robust to that perturbation, even when the images were perturbed by multiple perturbations simultaneously. The error rates for all classifiers of both datasets are reported as the average error rate among all 200 test sets.

## 3. Results

**Figure 5.**
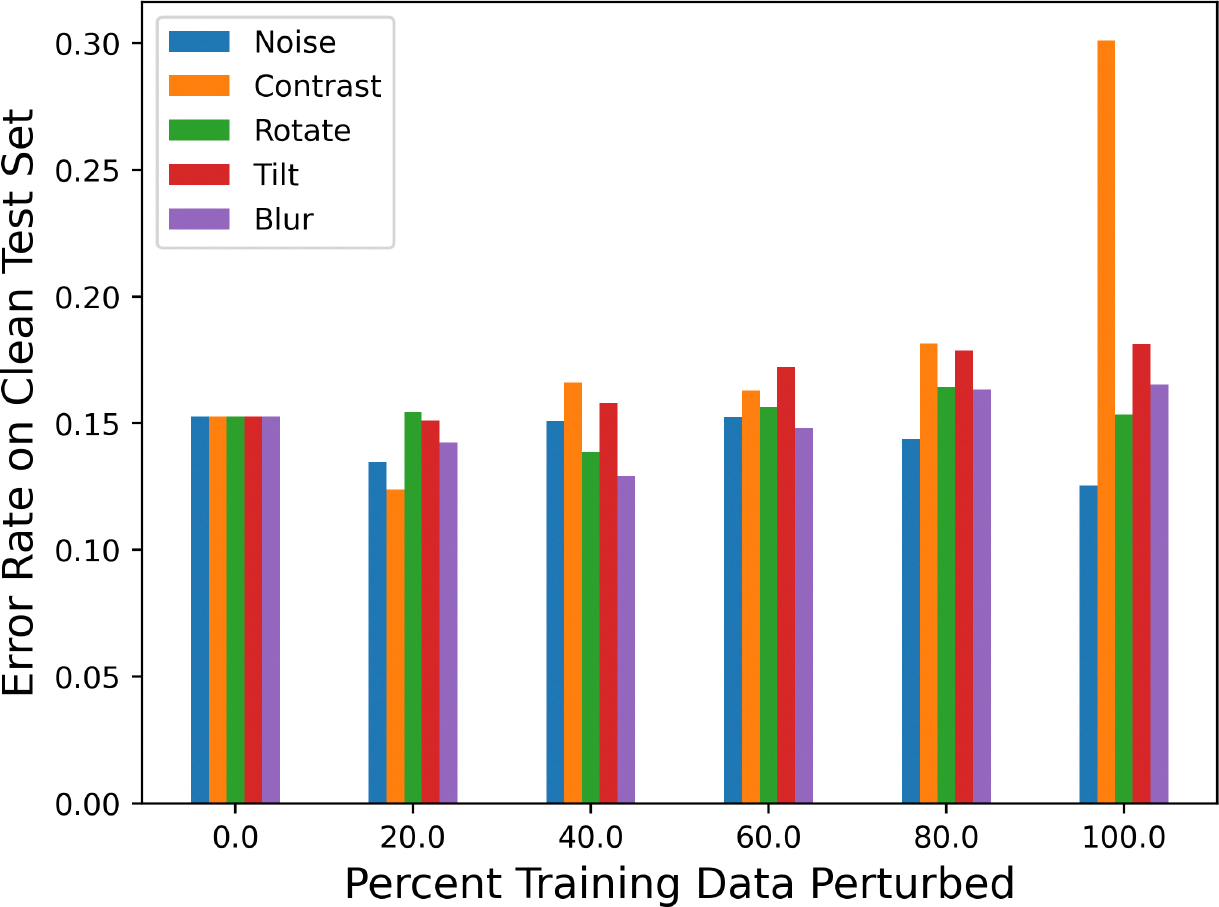
Performance of classifiers trained with perturbed training images on unperturbed test data. The horizontal axis shows the percent of training data perturbed. The legend displays which perturbation the classifier’s training set was perturbed by.

The first step of this work was to obtain the baseline performance of each classifier by using them to predict the unperturbed test set data. Figure 5 shows the error rate (percent of images incorrectly classified) of each classifier *f*_*p*_^*n*^ for all combinations of *p* and *n* (when *p* = 0; this is the same as the unperturbed classifier). The exact numeric results for this graph can be found in Table A1. The plotted error rates were averaged over 10 different neural network architectures. The unperturbed classifier achieved approximately a 15.3% error rate on the test data, which demonstrates that the classifier can learn the underlying data distribution. There was no noticeable decrease in the accuracy for the classifiers perturbed with 20% of the data (*f*_*20*_^*n*^). There was also a minimal increase in the error rate (<0.03) for all classifiers aside from those trained with 80–100% of the training images perturbed. Intuitively, this makes sense, because the classifier was mostly trained with perturbed data and was, thus, not learning as much of the unperturbed data distribution. Tables A2 and A3 contain the precision and recall of each classifier when predicting unperturbed data. Overall, the recall for all classifiers remained quite high (>0.9), while the precision was more varied (0.676–0.886), indicating that the classifiers had higher false positive rates than false negative rates on unperturbed data.

**Figure 6.**
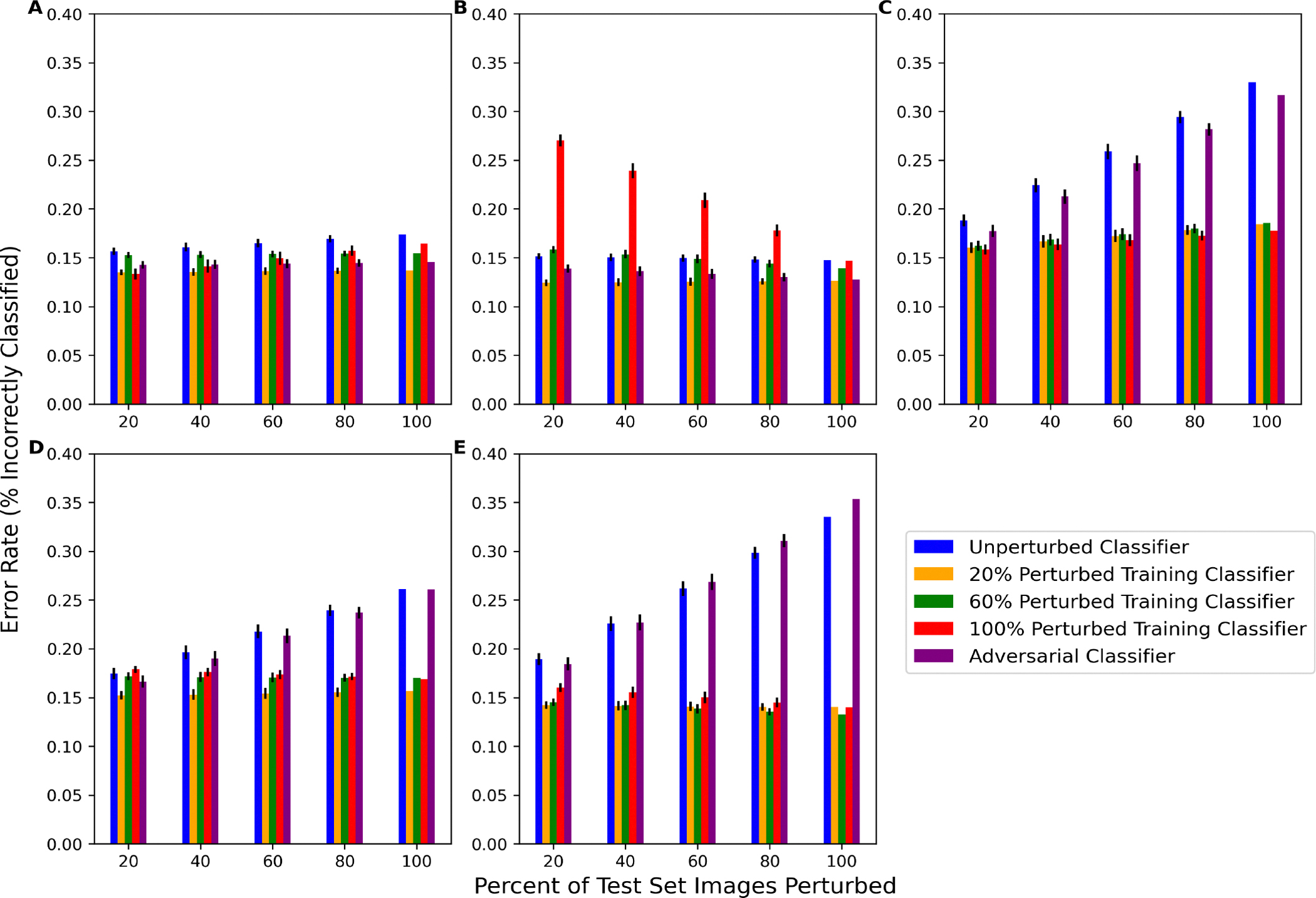
Performance of the classifier trained with unperturbed data (blue) and the performance of the classifier trained with 20% (orange), 60% (green), and 100% (red) perturbed images and 100% SPSA adversarial noise on test data with various amounts of perturbed data. Images are perturbed by (**A**) Gaussian noise, (**B**) contrast, (**C**) rotation, (**D**) tilt, and (**E**) blur. Error bars are the average of the error rate’s standard deviation for all 10 network architectures.

The behavior of classifiers on perturbed test set images was also analyzed for five classifier types: unperturbed, *f*_20_^*n*^ (classifier perturbed with 20% perturbed training images), *f*_60_^*n*^ (classifier perturbed with 60% perturbed training images), *f*_100_^*n*^ (classifier perturbed with 100% perturbed training images), and the SPSA adversarial classifier. As per Figure 4, for each of the 10 network architectures, classifiers were re-trained using only one of the perturbations at a time. The average error rates of the 10 deep classifier architectures for each classifier type on each perturbation are presented in Figure 6. The corresponding numeric data are presented in Table A4. The unperturbed classifier’s error rate increased as the percent of perturbed images in the test set increased for rotation, tilt, and blur and, to a lesser extent, Gaussian noise. One notable result was that the unperturbed classifier’s performance did not change much at any percentage of contrast-perturbed images. The adversarial classifier exhibited similar performance trends on the rotation-, tilt-, and blur-perturbed test data, indicating that the adversarial classifier was not capable of learning these perturbations. The adversarial classifier performed better than the unperturbed classifier on noise-perturbed and contrast-perturbed data, which may indicate similarities between SPSA adversarial noise and how these perturbations affect the data.

In addition to accuracy, it was also important to measure other metrics to obtain a more comprehensive view of the classifiers’ behavior. Precision and recall were also measured, and these data are presented in Tables A5 and A6. Overall, with more perturbed test data, the precision of the unperturbed classifier tended to decrease and the recall stayed high, which may indicate that the noise caused the classifier to favor negative samples over positive samples when perturbations were introduced. The changes in precision are reflective of the changes in the error rate, as the unperturbed classifier showed decreases in precision similar to its increases in the error rate for rotation-, tilt-, and blur-perturbed images. This is in contrast to the perturbed classifiers, where precision are affected less severely.

Each of the perturbed classifiers showed very little difference in performance from any of the test set scenarios, with the exception of the performance of *f*_100_^*n*^ on contrast-perturbed data. Otherwise, all of the perturbed classifiers mostly outperformed the unperturbed and adversarial classifiers. All perturbed classifiers except *f*_100_^*contrast*^, *f*_60_^*contrast*^, and *f*_60_^*noise*^ had a significantly lower error rate (better performance) than the unperturbed classifier for most amounts of perturbed test data. The specific *p*-values computed via the Wilcoxon Signed Rank test comparing the average performance of each of the 10 unperturbed classifiers to each of the 10 perturbed classifiers are presented in Table A7. These data show that, in addition to being able to perform well on the unperturbed test data, the performance also significantly improved on perturbed test data for most perturbed classifiers. Furthermore, there was little difference between the performance of each of the perturbed classifiers, demonstrating that there was not a linear relationship between the percentage of perturbed training images and the decrease in the performance of partially perturbed test sets. The computation of the average structural similarity index metric (SSIM) between unperturbed test sets and perturbed test sets also revealed that there was no linear relationship between perturbation intensity and classifier performance. These data are presented in Table A8. Essentially, while the blur perturbation had a more significant impact on classifier performance than noise or contrast, the average SSIM of blur-perturbed test set images was 0.827, whereas the noise and contrast were actually more intense perturbations with SSIMs of 0.678 and 0.632.

**Figure 7.**
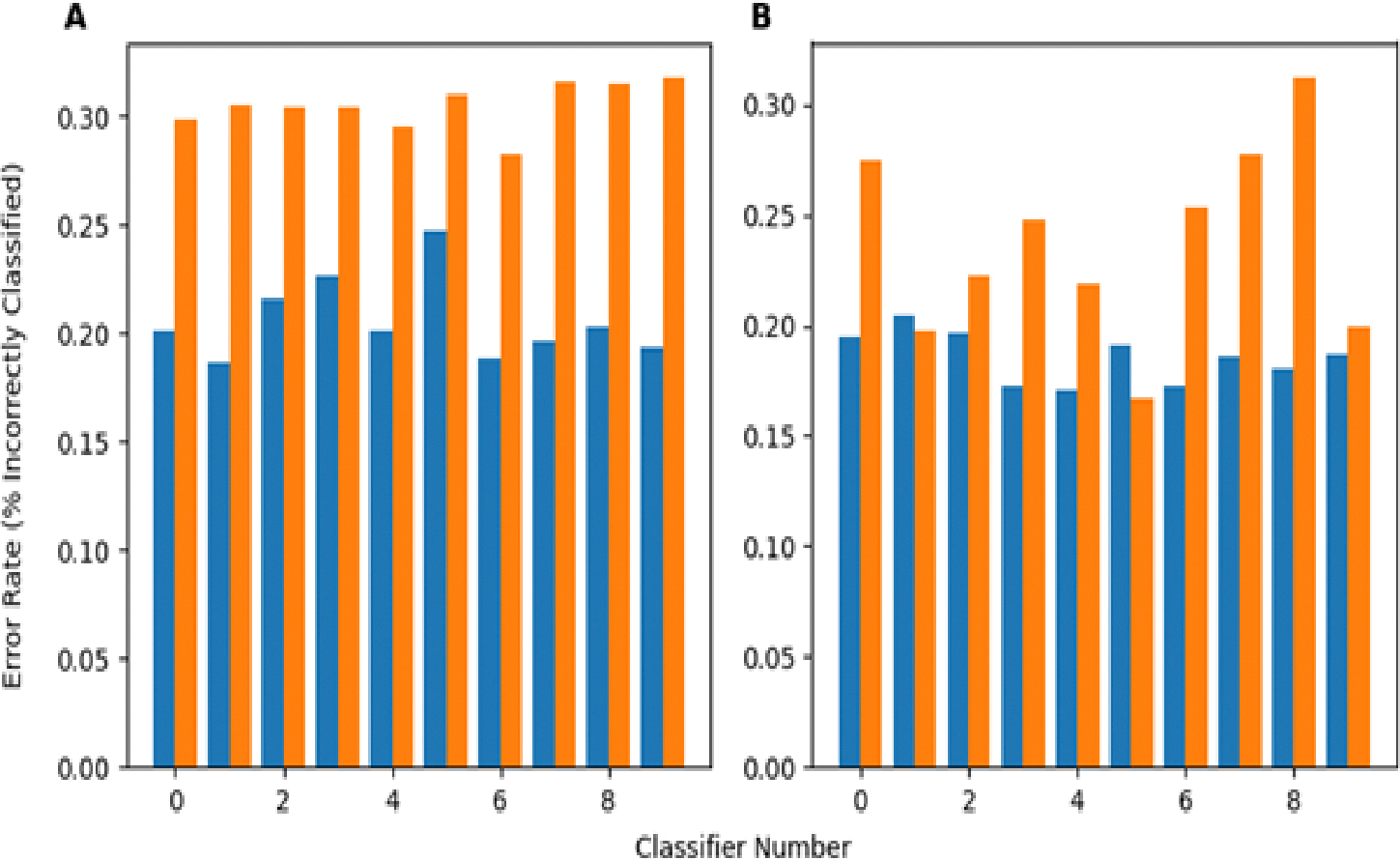
(**A**) (**left**) Average error rate of classifiers trained on the X-ray training data and training data partially perturbed by each perturbation on multiple/simultaneously randomly perturbed test images over 200 test sets. (**B**) (**right**) Average error rate of classifiers trained on the ultrasound training data and training data partially perturbed by each perturbation on multiple/simultaneously randomly perturbed test images over 200 test sets.

In a separate investigation, evaluating how training on perturbed datasets that simultaneously involved how multiple perturbations will affect the robustness of the developed classifier was also looked at. This task involved both images from the PneumoniaMNIST and, separately, from the BUSI dataset. This investigation was performed by training 10 neural networks (of different architectures) on the unperturbed training data for both the X-ray and ultrasound datasets (20 total). Then, 10% of the training dataset was perturbed by each of the five perturbations used in this study, leaving 50% unperturbed, and each classifier was re-trained (by shuffling the previous weights) with these data to obtain another 20 total classifiers trained using somewhat perturbed training data. For each test sample, each perturbation had a 50% chance of being chosen to be applied to that sample, meaning each sample could be perturbed by some, all, or none of the perturbations. Figure 7A shows the performance of the perturbed and unperturbed versions of classifiers from each network architecture trained on PneumoniaMNIST on this version of the perturbed dataset. The perturbed classifiers performed significantly better based on the Wilcoxon Signed Rank *p*-value (0.000977) between the perturbed classifier error rates and unperturbed classifier error rates. On average, the perturbed classifiers had an error rate 0.0986 lower, or an improvement of 9.86%, compared to their unperturbed counterparts for the PneumoniaMNIST dataset. Figure 7B shows this same behavior for the classifiers trained with BUSI, with an error rate decrease of 0.0515 between perturbed and unperturbed classifiers. Again, the perturbed classifiers performed significantly better (*p* = 0.00684) than their unperturbed counterparts on the BUSI data. The exact error rates of each classifier’s predictions on images subject to multiple perturbations is presented in Table A9. The error rates for each network architecture are included in Table A7.

## 4. Discussion

The main goal of this study was to investigate a procedure for obtaining robust medical imaging classifiers by using perturbations on both the training and test set images. Essentially, the stability of a classifier’s performance on both perturbed and unperturbed data was evaluated. The given classifier for this study was simulated by training a series of 10 convolutional neural networks on an example dataset. Each of these networks was then passed through the proposed procedure, and their error rates were averaged over all 10 networks.

Given a classifier *f* and a dataset, we evaluated the robustness of this classifier by comparing it to classifiers that were trained on perturbed training data where some percentage of images were perturbed i.e., where one would expect the classifier to perform well. In Figure 6C–E, the unperturbed classifier *f* performs worse (i.e., increases the error rate) as the percentage of test images that are perturbed increases. By perturbing even a small amount of the training set and re-training the classifier weights, significant improvements in classifier performance can be achieved on data perturbed by tilt, blur, and rotation. This implies that the original classifier was not learning information that would help it classify perturbed images, as even a small amount of perturbation in the training set resulted in noticeable improvements in performance. As such, this difference in performance between the perturbed classifier and the original classifier can be viewed as an indicator of the given classifier’s robustness (or lack thereof). Therefore, based on this procedure, the classifier *f* would not be considered robust to these perturbations. Conversely, although the classifier trained with Gaussian-noise-perturbed and contrast-perturbed data performed better than the unperturbed classifier, the original classifier still showed relatively high performance and did not decrease meaningfully in performance with respect to the percentage of test set images perturbed. While the perturbed classifier may technically be more robust than the given classifier to these perturbations, this demonstrates that the original classifier was still somewhat stable given changes in contrast and noise as it still performed well (maintains < 20% error rate). Ultimately, this shows that it is important to perturb some amount of both the training and test set images to generate an increase in robustness as a trade-off for a minimal loss in accuracy.

Another finding of this work is that a robust classifier can perform well on both perturbed and unperturbed test data. For example, while the error rate of the given classifier *f* was high in response to 60–100% rotation-, tilt-, and blur-perturbed test data, the error rate was still acceptable (<20%) with 20% of the test images perturbed. Therefore, it may be possible to determine just how much noise a classifier can handle before it is no longer useful. For instance, if 20% of the data are measured in a different clinic or using a different machine than the rest of the data, then the classifier may still perform reasonably. However, if half the data include this different distribution, it may not. Furthermore, the difference between the perturbed classifier’s performance and the unperturbed classifier’s performance was similar for each perturbation, as can be seen in Figure 6. However, as previously stated, as more of the data are perturbed, it is clear that the original classifier was much less robust to rotation, tilt, and blur perturbations than Gaussian noise and contrast. Therefore, a classifier may be robust to perturbations when only a small amount of the data are perturbed; however, it cannot be said whether this robustness will be maintained or diminish based on this one performance test.

If the sample size allows, perturbing a portion of data may improve the robustness of the classifier. For example, *f*_20_^*rotation*^ performed similarly on the unperturbed test set data to the unperturbed classifier *f*. Having comparable performance is understandable as 80% of the training data for *f*_20_^*rotation*^ were still unperturbed. Likewise, *f*_20_^*rotation*^ performed better than *f* no matter how much test data were perturbed (as long as some of them were perturbed). Additionally, and perhaps more importantly, the error rate of *f*_20_^*rotation*^ did not significantly increase no matter how many of the test images were perturbed, indicating that this classifier performed well and consistent with its performance. So, while *f* may only be robust to data with a few images perturbed by rotation, perturbing the data with just 20% rotated training images appeared to create a more robust classifier than the original/given classifier. While the variances of the performance between the two classifiers across the 200 randomly perturbed test sets were comparable, neither were particularly high and, therefore, did not call the robustness of either classifier into question.

Since perturbations are random in nature, most images will, to varying degrees, be affected by multiple perturbations. To test if training with perturbed data can improve classifier robustness to simultaneous/multiple perturbations, classifiers were generated for two datasets using the original training data, and then, subsequently, a new set of perturbed classifiers was trained using training data where 10% of the training set was perturbed by each perturbation (leaving 50% unperturbed). Perturbing the training data with sets of each perturbation improved classifier robustness to multiple perturbations, as measured by the error rate, with small images and a large sample size (Figure 7A) and larger images, but a smaller sample size (Figure 7B). While the classifiers trained on the BUSI data showed less of a performance improvement (error rate decrease) than the PneumoniaMNIST classifiers, both still showed significant decreases in the error rate when the training data of each classifier were perturbed, as is shown in Figure 7 and Table A7. The smaller performance increase with the BUSI-trained classifiers may be due to the larger number of parameters, leading the models to be more sensitive to noise, and the smaller sample size of the training dataset, making it more difficult for the classifiers to learn the underlying data distribution. In addition, since there was not much similarity between the 28 × 28 chest X-ray images and 500 × 500 breast ultrasound images, it is unlikely that the classifiers were performing better due to overfitting or some dataset-specific phenomenon. Despite these differences, this approach improved the performance of the classifiers for both datasets with different sizes and applications. This implies that this approach can improve the robustness of other, similar medical imaging datasets to common perturbations. Essentially, if a set of perturbations is known to possibly affect an image, a small portion of training set images should be transformed using those perturbations. Nevertheless, these results show that training classifiers with perturbed data from several individual perturbations seems to make the classifiers more robust even to multiple simultaneous perturbations.

To put our findings into a broader context, comparisons with adversarial training were also performed. Adversarial training approaches have notable strengths, as they are the de facto best method for complex adversaries that may not be visually perceptible. They have also been shown to improve the robustness of classifiers in many different applications. Additionally, they have wider applicability by being able to be used in models like generative adversarial networks for applications such as image denoising. However, the general weaknesses of adversarial classifiers have been shown to not be able to generalize well, struggle with multiple sources of adversarial noise, and be computationally complex. Although SPSA is only one type of adversarial training, these broad weaknesses make it unlikely that implementing other forms of adversarial noise would improve robustness in this scenario. It is important to note that adversarial training can be limited in terms of which types of adversaries it can be applied to [49]. For instance, the SPSA-trained classifier showed the same general trends in performance as the unperturbed classifier when applied to rotation-, tilt-, and blur-perturbed test data. This indicates a lack of robustness to these perturbations, which is expected since rotation, tilt, and blur affect image data differently than SPSA. Moreover, previous studies have shown that perturbing data with certain types of untargeted noise can create similar changes in performance to adversarial training [50]. There is also not much variance in the computational complexity of each perturbation, whereas the computational requirement of adversarial training can vary significantly. More details on the computational complexity of this work is contained in Tables A10 and A11. It was also demonstrated in this study that perturbing the training data can result in classifiers being robust to multiple perturbations, a notable area that adversarial models are weak in. While adversarial attacks can prepare classifiers for certain perturbations, if a specific perturbation is suspected of being possibly present in a dataset, especially if multiple perturbations are suspected, a dataset should be trained with a small number of images augmented using that perturbation.

As the previous results have shown that the approach in this study could evaluate and improve the robustness of a series of deep classifiers, future implementations of this work should continue to vary the amount of perturbed training samples to develop a robust classifier. Previous research has established the idea that classifiers robust to a perturbation should perform similarly to classifiers trained with a small amount of that perturbation [51]. To find this small amount, it is important to thoroughly vary the amount of perturbed images in the training and test set, as was performed in this study. While the experiments in this study were performed on simulated perturbations, real-world diagnostic workflows could prepare their classifiers for noise by preemptively diversifying their training set with expected perturbations. Although this cannot circumvent the regulatory need for clinical validation tests, this approach can indicate which diagnostic classifiers are likely to be robust and, also, a liberal estimation of how robust the classifier can be.

### Limitations

There are several limitations to this work that should be investigated in future studies. First, the severity of each of the perturbations was fixed for all applications in this study. In real-world diagnostic applications, noise may not be present in images at the same severity. While the SSIM data in Table A8 imply that perturbation intensity is not directly related to robustness, future studies should investigate how robust classifiers are when the intensity/severity of each individual perturbation is varied.

Another limitation of this study is that it is unknown if this robustness will occur for all types of deep learning classifiers. For example, it is difficult to interpret exactly why a classifier is better at predicting certain images than others, as most neural networks have many trainable weights that must be accounted for. Additionally, although this study showed robustness for networks trained on grayscale images, other image modalities produce three-channel (RGB) or three-dimensional images that may require more complex classifiers. As such, future studies should investigate the impact of classifier complexity on its robustness.

One final consideration is that, while this procedure can improve the robustness of classifiers in certain scenarios, it will not necessarily create a clinic-ready classifier. Just as the adversarially trained classifier did not perform well on the images augmented using the perturbations in the study, these perturbations are not the only sources of noise an image may be subjected to. Testing a classifier under more diverse perturbations would require more iterations and, therefore, be much more computationally expensive and infeasible for most clinical applications. Furthermore, the clinical application of the classifiers involved in this study cannot be ensured without actual validation on new clinical data, which should be performed in future studies.

## 5. Conclusions

This work presented a detailed study of how perturbations in the training and/or test set can affect a classifier’s robustness based on two applications. To this end, a series of convolutional neural networks was trained using both perturbed and unperturbed data.

First, the baseline robustness of a given classifier, i.e., the classifiers trained with completely unperturbed data, was evaluated by comparing the performance of the classifier on unperturbed test data to the performance of classifiers trained with some percentage of perturbed training data on unperturbed test data. On average, it was found that perturbing even most of the training data did not cause a significant performance decrease when evaluating a classifier on the unperturbed test data. This is important because it indicates that a perturbed classifier can perform similarly to the original classifier on unperturbed data while potentially providing better robustness on perturbed data.

Furthermore, the original classifiers were tested on test data with different amounts of samples perturbed (20%, 40%, 60%, 80%, 100%). The performance of the original classifiers was then compared to classifiers trained with a low (20%), medium (60%), and high (100%) number of perturbed samples, as well as a classifier trained with adversarial data. This investigation showed that classifiers that were trained with small perturbations seemed to provide a good trade-off between a minor loss of accuracy when predicting unperturbed data and the robustness gained for data that included perturbations compared to the classifier trained on the unperturbed data. This was even the case in most applications when the perturbations that the classifiers were tested on were larger than those used for their training. Furthermore, training the classifiers on data where parts of the data had been perturbed by different individual perturbations seemed to also result in improved robustness when combinations of these perturbations were present in the test set. Ultimately, this work demonstrated the importance of perturbing both the training samples and the test samples simultaneously to generate robust classifiers based on medical imaging data.

## Supplementary Material

Supplementary_including_Tables_S1_and_S2

**Supplementary Materials:** The following supporting information can be downloaded at: https://www.mdpi.com/article/10.3390/biomedinformatics4020050/s1, Table S1: A table of the neural network architectures of each of the 10 classifiers trained on the PneumoniaMNIST dataset. The layers of each architecture are sequential and the dimensions of each layer are contained parenthetically next to each layer type; Table S2: A table of the neural network architectures of each of the 10 classifiers trained on the BUSI dataset. The layers of each architecture are sequential and the dimensions of each layer are contained parenthetically next to each layer type.

## Figures and Tables

**Table 1.** Brief description of each type of perturbation and the corresponding severities.

Perturbation Type (*n*)	Description	Severity
Gaussian Noise	Adding or subtracting a value randomly selected from N (0, s^2^)	s = 0.08, randomly selected for each pixel
Contrast	Adjust pixel values closer to the image maximum and minimum	Enhancement factor (EF) = 3.3 (EF = 1 is the original image)
Rotation	Turn image by a certain angle *θ* about the xy plane	*θ* randomly selected integer between −90 and 90 degrees for each image
Tilt	Turn image by a certain angle *θ* about the xy plane	*θ* randomly selected integer between 0 and 90 degrees for each image
Blur	Defocus blur using disk kernel, function hyperparameters defined in [17]	Kernel size = 3 Kernel StDev = 0.1

## Data Availability

The datasets analyzed during the current study are available in the MedMNIST repository, https://medmnist.com/ (accessed on 3 February 2023), or are included in the published article found here: https://www.sciencedirect.com/science/article/pii/S2352340919312181 (accessed on 20 July 2023). The software used in this study requires Python 3.0 or newer and can be found at https://github.rpi.edu/Hahn-Research-Group/PerturbRobust (accessed on 5 February 2024).

## Appendix A

### Appendix A.1

This Appendix contains the numerical data supporting each of the figures in the results. Furthermore, the precision and recall scores are included to support the analysis of the average false positive rate and false negative rate of the classifiers included in this study.

**Table A1.** Average error rates of the perturbed classifiers on the unperturbed test set data.

Perturbation	0% Training Data Perturbed	20% Training Data Perturbed	40% Training Data Perturbed	60% Training Data Perturbed	100% Training Data Perturbed

Noise	0.153	0.134	0.151	0.152	0.125
Contrast	0.153	0.124	0.166	0.162	0.301
Rotation	0.153	0.154	0.138	0.157	0.154
Tilt	0.153	0.151	0.158	0.172	0.181
Blur	0.153	0.142	0.129	0.148	0.165

**Table A2.** Average precision scores of the perturbed classifiers on unperturbed test set data.

Perturbation	0% Training Data Perturbed	20% Training Data Perturbed	40% Training Data Perturbed	60% Training Data Perturbed	100% Training Data Perturbed

Noise	0.807	0.836	0.816	0.812	0.886
Contrast	0.807	0.845	0.801	0.800	0.676
Rotation	0.807	0.811	0.837	0.810	0.825
Tilt	0.807	0.817	0.810	0.791	0.787
Blur	0.807	0.825	0.840	0.821	0.843

**Table A3.** Average recall scores of the perturbed classifiers on unperturbed test set data.

Perturbation	0% Training Data Perturbed	20% Training Data Perturbed	40% Training Data Perturbed	60% Training Data Perturbed	100% Training Data Perturbed

Noise	0.995	0.977	0.981	0.970	0.920
Contrast	0.995	0.985	0.991	0.993	0.998
Rotation	0.995	0.985	0.970	0.978	0.961
Tilt	0.995	0.982	0.981	0.985	0.979
Blur	0.995	0.982	0.981	0.975	0.910

**Table A4.** Average error rates of each classifier on different amounts of perturbed test data. The error rates were averaged across classifiers trained with each of the 10 different network architectures described in Table S1.

Type/Amount of Test Set Perturbation	Unperturbed Classifier	20% Perturbed Classifier	60% Perturbed Classifier	100% Perturbed Classifier	Adversarial Classifier

Noise/20%	0.157	0.135	0.153	0.133	0.143
Noise/40%	0.161	0.136	0.153	0.141	0.143
Noise/60%	0.165	0.136	0.154	0.149	0.144
Noise/80%	0.169	0.137	0.154	0.157	0.145
Noise/100%	0.173	0.137	0.155	0.165	0.145
Contrast/20%	0.151	0.124	0.158	0.270	0.139
Contrast/40%	0.151	0.125	0.154	0.239	0.136
Contrast/60%	0.150	0.125	0.149	0.209	0.133
Contrast/80%	0.149	0.126	0.144	0.178	0.130
Contrast/100%	0.147	0.126	0.139	0.147	0.128
Rotate/20%	0.188	0.160	0.162	0.158	0.177
Rotate/40%	0.224	0.167	0.169	0.164	0.213
Rotate/60%	0.259	0.172	0.174	0.168	0.247
Rotate/80%	0.294	0.178	0.180	0.173	0.282
Rotate/100%	0.330	0.184	0.186	0.177	0.317
Tilt/20%	0.175	0.152	0.172	0.179	0.166
Tilt/40%	0.196	0.153	0.171	0.176	0.190
Tilt/60%	0.218	0.154	0.170	0.174	0.213
Tilt/80%	0.239	0.155	0.170	0.171	0.237
Tilt/100%	0.261	0.157	0.170	0.169	0.261
Blur/20%	0.189	0.142	0.145	0.160	0.184
Blur/40%	0.226	0.142	0.142	0.155	0.227
Blur/60%	0.261	0.141	0.139	0.150	0.268
Blur/80%	0.298	0.140	0.136	0.145	0.311
Blur/100%	0.335	0.140	0.133	0.140	0.353

**Table A5.** Average precision of each classifier on different amounts of perturbed test data. The precision scores were averaged across classifiers trained with each of the 10 different network architectures described in Table S1.

Type/Amount of Test Set Perturbation	Unperturbed Classifier	20% Perturbed Classifier	60% Perturbed Classifier	100% Perturbed Classifier	Adversarial Classifier

Noise/20%	0.803	0.837	0.812	0.866	0.822
Noise/40%	0.799	0.837	0.812	0.847	0.823
Noise/60%	0.795	0.837	0.811	0.830	0.825
Noise/80%	0.792	0.837	0.811	0.814	0.826
Noise/100%	0.788	0.837	0.811	0.799	0.828
Contrast/20%	0.809	0.847	0.805	0.700	0.827
Contrast/40%	0.810	0.849	0.811	0.727	0.834
Contrast/60%	0.812	0.851	0.817	0.755	0.841
Contrast/80%	0.814	0.853	0.824	0.786	0.849
Contrast/100%	0.816	0.856	0.830	0.820	0.857
Rotate/20%	0.771	0.805	0.804	0.819	0.783
Rotate/40%	0.738	0.799	0.798	0.812	0.749
Rotate/60%	0.708	0.794	0.793	0.806	0.719
Rotate/80%	0.681	0.789	0.787	0.801	0.691
Rotate/100%	0.655	0.785	0.782	0.795	0.665
Tilt/20%	0.784	0.818	0.793	0.790	0.797
Tilt/40%	0.764	0.820	0.795	0.793	0.775
Tilt/60%	0.745	0.821	0.797	0.796	0.755
Tilt/80%	0.726	0.823	0.799	0.799	0.735
Tilt/100%	0.708	0.825	0.801	0.802	0.717
Blur/20%	0.770	0.826	0.825	0.840	0.776
Blur/40%	0.736	0.828	0.829	0.837	0.736
Blur/60%	0.706	0.830	0.833	0.835	0.701
Blur/80%	0.678	0.832	0.837	0.833	0.669
Blur/100%	0.652	0.834	0.841	0.830	0.639

**Table A6.** Average recall of each classifier on different amounts of perturbed test data. The recall scores were averaged across classifiers trained with each of the 10 different network architectures described in Table S1.

Type/Amount of Test Set Perturbation	Unperturbed Classifier	20% Perturbed Classifier	60% Perturbed Classifier	100% Perturbed Classifier	Adversarial Classifier

Noise/20%	0.994	0.976	0.986	0.933	0.986
Noise/40%	0.993	0.975	0.985	0.946	0.983
Noise/60%	0.993	0.973	0.985	0.960	0.979
Noise/80%	0.992	0.972	0.985	0.973	0.976
Noise/100%	0.991	0.971	0.984	0.987	0.972
Contrast/20%	0.993	0.981	0.986	0.996	0.984
Contrast/40%	0.992	0.976	0.985	0.993	0.977
Contrast/60%	0.991	0.972	0.984	0.990	0.971
Contrast/80%	0.989	0.968	0.982	0.987	0.965
Contrast/100%	0.988	0.963	0.981	0.984	0.958
Rotate/20%	0.995	0.983	0.981	0.963	0.991
Rotate/40%	0.996	0.982	0.981	0.966	0.992
Rotate/60%	0.997	0.980	0.981	0.968	0.994
Rotate/80%	0.997	0.978	0.980	0.970	0.995
Rotate/100%	0.998	0.976	0.980	0.973	0.997
Tilt/20%	0.994	0.978	0.983	0.978	0.986
Tilt/40%	0.993	0.973	0.980	0.976	0.983
Tilt/60%	0.993	0.968	0.978	0.975	0.980
Tilt/80%	0.992	0.962	0.975	0.973	0.977
Tilt/100%	0.991	0.957	0.971	0.971	0.973
Blur/20%	0.996	0.980	0.977	0.923	0.992
Blur/40%	0.997	0.978	0.976	0.937	0.994
Blur/60%	0.998	0.976	0.976	0.950	0.996
Blur/80%	0.999	0.974	0.975	0.964	0.998
Blur/100%	1.000	0.972	0.974	0.978	1.000

**Table A7.** Wilcoxon Signed Rank computed *p*-values between the 10 unperturbed classifier’s and 10 perturbed classifiers’ average performance on 200 perturbed test sets for each perturbation.

Type/Amount of Test Set Perturbation	20% Perturbed Classifier	60% Perturbed Classifier	100% Perturbed Classifier	Adversarial Classifier

Noise/20%	0.0186	0.278	0.00488	0.0527
Noise/40%	0.0244	0.246	0.0186	0.0420
Noise/60%	0.0186	0.188	0.0801	0.0527
Noise/80%	0.0186	0.188	0.161	0.0801
Noise/100%	0.0186	0.188	0.246	0.116
Contrast/20%	0.00488	0.884	1.00	0.0801
Contrast/40%	0.00293	0.688	1.00	0.322
Contrast/60%	0.00293	0.423	1.00	0.0137
Contrast/80%	0.000977	0.313	0.990	0.00977
Contrast/100%	0.00488	0.157	0.216	0.00977
Rotate/20%	0.00977	0.00684	0.00488	0.0967
Rotate/40%	0.000977	0.000977	0.000977	0.801
Rotate/60%	0.000977	0.000977	0.000977	0.0967
Rotate/80%	0.000977	0.000977	0.000977	0.138
Rotate/100%	0.000977	0.000977	0.000977	0.116
Tilt/20%	0.138	0.385	0.983	0.188
Tilt/40%	0.00293	0.00977	0.539	0.278
Tilt/60%	0.000977	0.00195	0.0244	0.423
Tilt/80%	0.000977	0.000977	0.00977	0.423
Tilt/100%	0.000977	0.000977	0.00977	0.577
Blur/20%	0.000977	0.000977	0.00683	0.313
Blur/40%	0.000977	0.000977	0.000977	0.577
Blur/60%	0.000977	0.000977	0.000977	0.615
Blur/80%	0.000977	0.000977	0.000977	0.839
Blur/100%	0.000977	0.000977	0.000977	0.958

**Table A8.** Average structural similarity index metric (SSIM) and standard deviation of the average structural similarity index metric among 200 test sets, where all images were perturbed by noise, contrast, rotation, tilt, blur, or multiple perturbations.

Perturbation	Average SSIM	SSIM Stdev.

Noise	0.678	0.126
Contrast	0.632	0.0272
Rotate	0.325	0.273
Tilt	0.524	0.311
Blur	0.827	0.0466
Multi	0.395	0.0104

**Table A9.** Error rate of unperturbed and perturbed classifiers from each neural network architecture on test set images subject to multiple random perturbations. Unperturbed classifiers were trained with their respective training set images (PneumoniaMNIST or BUSI), while perturbed classifiers were trained with data that were 50% unperturbed, and 10% were perturbed by each perturbation.

Classifier Number (Dataset)	Unperturbed Training Performance	Perturbed Training Performance	Error Rate Change

1 (PneumoniaMNIST)	0.202	0.298	0.096
2 (PneumoniaMNIST)	0.186	0.305	0.119
3 (PneumoniaMNIST)	0.216	0.304	0.088
4 (PneumoniaMNIST)	0.227	0.304	0.077
5 (PneumoniaMNIST)	0.201	0.296	0.095
6 (PneumoniaMNIST)	0.248	0.310	0.062
7 (PneumoniaMNIST)	0.189	0.283	0.094
8 (PneumoniaMNIST)	0.197	0.316	0.119
9 (PneumoniaMNIST)	0.204	0.316	0.112
10 (PneumoniaMNIST)	0.194	0.318	0.124
1 (BUSI)	0.195	0.275	0.080
2 (BUSI)	0.205	0.198	−0.007
3 (BUSI)	0.197	0.223	0.026
4 (BUSI)	0.173	0.248	0.075
5 (BUSI)	0.171	0.219	0.048
6 (BUSI)	0.191	0.167	−0.024
7 (BUSI)	0.172	0.253	0.081
8 (BUSI)	0.186	0.278	0.092
9 (BUSI)	0.181	0.312	0.131
10 (BUSI)	0.187	0.200	0.013

**Table A10.** Time taken to create 200 perturbed test sets with different percentages of perturbed data, all measured in seconds.

Perturbation	20%	40%	60%	80%	100%

Noise	3.60	5.55	7.30	9.25	10.95
Contrast	5.53	9.64	13.49	17.21	20.81
Rotation	8.51	13.34	17.29	21.39	25.78
Tilt	9.76	16.72	21.25	26.70	32.16
Blur	9.12	18.72	24.83	33.95	42.85

**Table A11.** Baseline time to test each network architecture on unperturbed data, measured in seconds. Time was measured by taking the average time it took each classifier to predict the training data 10 times.

Network Architecture	Average Time to Test (s)

1 (PneumoniaMNIST)	1.92
2 (PneumoniaMNIST)	0.810
3 (PneumoniaMNIST)	0.748
4 (PneumoniaMNIST)	0.500
5 (PneumoniaMNIST)	0.820
6 (PneumoniaMNIST)	0.803
7 (PneumoniaMNIST)	1.07
8 (PneumoniaMNIST)	2.91
9 (PneumoniaMNIST)	1.46
10 (PneumoniaMNIST)	1.58

## Abbreviations

The following abbreviations are used in this manuscript:

ML: machine learning
AI: artificial intelligence
GAN: generative adversarial network
PDT: pixel-deflecting transform
NLCE: non-linear context encoder
BUSI: Breast Ultrasound Images
CT: computed tomography
MRI: magnetic resonance imaging
CAD: computer-aided diagnostics
SPSA: simultaneous perturbation stochastic approximation
