# Supplementary material for: Towards the Generation of Medical Imaging Classifiers Robust to Common Perturbations
Source: BioMedInformatics. Author manuscript; Available in PMC 2025 Jun 18. (PMC12176414; doi:10.3390/biomedinformatics4020050)
Supplement: Supplementary_including_Tables_S1_and_S2 [file NIHMS2085300-supplement-Supplementary_including_Tables_S1_and_S2.pdf]

Supplemental Information for: Towards the Generation of Medical Imaging Classifiers Robust to Common Perturbations

Joshua Chuah, Pingkun Yan, Ge Wang, Juergen Hahn

January 2024

The purpose of this supplemental document is to provide the exact architecture of the neural networks used in this study. Each neural network was generated by the AutoKeras neural architecture search algorithm. For each dataset, each of the 10 classifiers was generated by iterating 20 times over potential neural network architectures until the one with the best performance was chosen. Different random seeds were used to initialize the algorithm for each of the 10 runs per dataset (the first proposed architecture was different in each run).

Each classifier is a variant of a convolutional neural network, which aims to compress the original images into feature maps that the classifier uses to distinguish between classes. The last dimension of the Conv2D or SeparableConv2D layers are the number of feature maps used in each layer. A list of the potential layers that can be used in a Keras model can be found at https://keras.io/api/layers/.

Classifier Number	Layers between Input Set and Output (Dim.)
1	Conv2D(1x24x24x32)<br>Conv2D (1x20x20x32)<br>MaxPooling2D (1x5x5x32)<br>Conv2D(1x5x5x256)<br>Conv2D(1x5x5x32)<br>MaxPooling2D(1x2x2x32)<br>Flatten(1x128)<br>Dropout (1x128)<br>Dense (1x1)
2	Conv2D (1x26x26x32)<br>Conv2D (1x24x24x32)<br>MaxPooling2D (1x12x12x32)<br>Conv2D(1x10x10x256)<br>Conv2D(1x8x8x32)<br>MaxPooling2D(1x4x4x32)

	Flatten(1x512)<br>Dropout (1x512)<br>Dense (1x1)
3	Conv2D (1x22x22x128)<br>MaxPooling2D (1x3x3x128)<br>Conv2D(1x3x3x32)<br>MaxPooling2D(1x1x1x32)<br>Flatten(1x32)<br>Dense (1x1)
4	Conv2D (1x24x24x32)<br>MaxPooling2D (1x6x6x32)<br>Conv2D(1x6x6x32)<br>MaxPooling2D (1x2x2x32)<br>Conv2D(1x2x2x32)<br>MaxPooling2D(1x1x1x32)<br>Flatten(1x32)<br>Dropout (1x32)<br>Dense (1x1)
5	Conv2D (1x26x26x32)<br>Conv2D (1x24x24x32)<br>MaxPooling2D (1x12x12x32)<br>Conv2D(1x10x10x256)<br>Conv2D(1x8x8x32)<br>MaxPooling2D(1x4x4x32)<br>Conv2D(1x4x4x16)<br>Conv2D(1x4x4x32)<br>MaxPooling2D(1x2x2x32)<br>GlobalAveragePooling2D(1x32)<br>Dropout (1x32)<br>Dense (1x1)
6	Conv2D (1x26x26x32)<br>Conv2D (1x24x24x32)<br>MaxPooling2D (1x12x12x32)<br>Conv2D(1x10x10x256)<br>Conv2D(1x8x8x32)<br>MaxPooling2D(1x4x4x32)<br>Conv2D(1x4x4x16)<br>Conv2D(1x4x4x32)<br>MaxPooling2D(1x2x2x32)<br>Flatten(1x32)<br>Dropout (1x32)<br>Dense (1x1)
7	Conv2D (1x24x24x32)<br>Conv2D (1x20x20x32)<br>MaxPooling2D (1x5x5x32)

	Conv2D(1x5x5x32)<br>Conv2D(1x5x5x32)<br>MaxPooling2D(1x2x2x32)<br>GlobalAveragePooling2D(1x32)<br>Dense (1x1)
8	Conv2D (1x26x26x32)<br>Conv2D (1x24x24x256)<br>MaxPooling2D (1x12x12x256)<br>Conv2D(1x10x10x32)<br>Conv2D(1x8x8x32)<br>MaxPooling2D(1x4x4x32)<br>GlobalAveragePooling2D(1x32)<br>Dense (1x1)
9	Conv2D (1x24x24x32)<br>Conv2D (1x20x20x32)<br>MaxPooling2D (1x5x5x32)<br>Conv2D(1x5x5x32)<br>Conv2D(1x5x5x32)<br>MaxPooling2D(1x2x2x32)<br>Flatten(1x128)<br>Dense (1x1)
10	Conv2D (1x24x24x32)<br>Conv2D (1x20x20x32)<br>MaxPooling2D (1x5x5x32)<br>Dropout(1x5x5x32)<br>Conv2D(1x5x5x256)<br>Conv2D(1x5x5x32)<br>MaxPooling2D(1x2x2x32)<br>Dropout(1x2x2x32)<br>GlobalAveragePooling2D(1x32)<br>Dense (1x1)

Table 1: S.1: A table of the neural network architectures of each of the 10 classifiers trained on the PneumoniaMNIST dataset. The layers of each architecture are sequential and the dimensions of each layer are contained parenthetically next to each layer type.

**Classifier Number**	**Layers between Input Set and Output (Dim.)**
1	Conv2D (1x498x498x64)<br>MaxPooling2D (1x249x249x64)<br>Conv2D(1x247x247x32)<br>MaxPooling2D(1x123x123x32)<br>Conv2D(1x121x121x256)<br>MaxPooling2D(1x60x60x256)<br>Flatten(1x921600)

	Dense (1x1)
2	Conv2D (1x498x498x32)
	Conv2D (1x498x498x64)
	MaxPooling2D (1x248x248x64)
	Conv2D(1x246x246x32)
	Conv2D(1x244x244x32)
	MaxPooling2D(1x122x122x32)
	Flatten(1x476288)
	Dense (1x1)
3	Conv2D (1x498x498x32)
	Conv2D (1x498x498x64)
	MaxPooling2D (1x248x248x64)
	Conv2D(1x246x246x32)
	Conv2D(1x244x244x32)
	MaxPooling2D(1x122x122x32)
	Flatten(1x476288)
	Dropout(1x476288)
	Dense (1x1)
4	SeparableConv2D(1x496x496x32)
	MaxPooling2D(1x124x124x32)
	SeparableConv2D(1x120x120x32)
	MaxPooling2D(1x30x30x32)
	Flatten(1x28800)
	Dense(1x1)
5	Conv2D (1x498x498x32)
	Conv2D (1x498x498x32)
	MaxPooling2D (1x248x248x32)
	Conv2D(1x246x246x32)
	Conv2D(1x244x244x32)
	MaxPooling2D(1x122x122x32)
	Flatten(1x476288)
	Dense (1x1)
6	Conv2D (1x498x498x64)
	Conv2D (1x498x498x32)
	MaxPooling2D (1x248x248x32)
	Conv2D(1x246x246x32)
	Conv2D(1x244x244x64)
	MaxPooling2D(1x122x122x64)
	Flatten(1x952576)
	Dense (1x1)
7	SeparableConv2D(1x496x496x32)
	MaxPooling2D(1x124x124x32)
	SeparableConv2D(1x120x120x32)
	MaxPooling2D(1x30x30x32)
	Flatten(1x28800)

	Dense(1x1)
8	Conv2D (1x498x498x32)<br>MaxPooling2D (1x249x249x32)<br>Conv2D(1x247x247x256)<br>MaxPooling2D(1x123x123x256)<br>Flatten(1x3873024)<br>Dense (1x1)
9	Conv2D (1x498x498x32)<br>Conv2D (1x498x498x32)<br>MaxPooling2D (1x248x248x32)<br>Dropout(1x248x248x32)<br>Conv2D(1x246x246x32)<br>Conv2D(1x244x244x32)<br>MaxPooling2D(1x122x122x32)<br>Dropout(1x122x122x32)<br>Flatten(1x476288)<br>Dense (1x1)
10	Conv2D (1x498x498x32)<br>MaxPooling2D (1x249x249x32)<br>Dropout(1x247x247x32)<br>MaxPooling2D(1x123x123x32)<br>Flatten(1x484128)<br>Dropout(1x484128)<br>Dense(1x1)

Table 2: S.2: A table of the neural network architectures of each of the 10 classifiers trained on the BUSI dataset. The layers of each architecture are sequential and the dimensions of each layer are contained parenthetically next to each layer type.
